# Experience of nurses regarding the clinical mentoring of student nurses in resource-limited settings

**DOI:** 10.4102/hsag.v25i0.1434

**Published:** 2020-08-06

**Authors:** Dhunraj Foolchand, Jeanette E. Maritz

**Affiliations:** 1 Department of Health Studies, Faculty of Human Sciences, University of South Africa, Pretoria, South Africa

**Keywords:** Clinical mentoring, Nursing student, Resource-limited, Developing, Support

## Abstract

**Background:**

One of the major challenges associated with nursing education in this 21st century is the practice preparation of student nurses to serve in complex healthcare environments and to ensure their fitness to practise in these environments. Clinical mentoring has shown promise in providing clinical learning support for student nurses. Most approaches are, however, biased towards higher-income settings without giving due consideration to the resources, culture and structures of health systems in resource-limited settings. It is also unclear how qualified nurses who act as nurse teachers experience the clinical mentoring of student nurses in resource-limited settings.

**Aim:**

This study aimed to explore and describe the experiences of qualified nurses regarding the clinical mentoring of nursing students in resource-limited settings.

**Setting:**

The study took place in Mauritius, a developing country.

**Methods:**

A qualitative, exploratory, descriptive approach was used with a purposive sample of eight nurses. Data were collected through face-to-face interviews and thematically analysed.

**Results:**

The findings indicated that mentoring *per se* was not practised, but rather general support, supervision or coaching. This resulted in the practice being less effective for its original purpose. Possible explanations included a lack of policy directives. Additionally, the mentoring practice was informal with unclear role expectations. Poor material and personal resources further compounded the challenges. An absence of buy-in and involvement of management along with a lack of monitoring clinical mentoring by the nursing school concludes the picture.

**Conclusion:**

Effective clinical mentoring requires an understanding of the mentoring process from a broader perspective. Mentors should be equipped with core competencies. Successful mentoring outcomes are dependent on a conducive clinical learning environment and a clear mentoring approach.

## Introduction

One of the major challenges associated with nursing education in the 21st century is the practice preparation of student nurses to serve in complex healthcare environments and to ensure that they are fit to practise in these environments (Mannino & Cotter 2016:1). The challenges are amplified in developing countries (also known as low- and middle-income countries) as these environments face a crisis in human resources, inadequate training of healthcare workers, lack of formal and developmental opportunities for the health workforce, a maldistribution of staff favouring urban areas, a skill-mix imbalance and high patient ratios (Schwerdtle, Morphet & Hall 2017:76). The above-mentioned aspects hamper developing countries’ abilities to reach the targets outlined by the Sustainable Development Goals (United Nations Department of Economic and Social Affairs 2018). Despite major transformation in the nursing profession over the last two decades (Caputi 2017:1), nursing education and training have not kept abreast of those rapid changes, particularly in poorly resourced developing countries (Bvumbwe & Mtshali 2018:1).

Mentoring has become an innovative tool to address learning and support for nursing students and nurses alike (Matin 2017:1; Sambunjak 2015:47). Matin (2017:1) broadly defines mentoring as the guiding, counselling, teaching and sharing of experiences to a mentee. Mentoring also implies a relationship between a more experienced person (mentor) and a younger person (mentee) (McMahon 2016:2). Clinical mentoring ensures that students are equipped with clinical practice skills that aim to address the theory–practice gap (Arnesson & Albinsson 2017:202).

The term ‘mentoring’ is multifaceted and can therefore easily be linked to *assessing, supervising, preceptoring* and *coaching*. All these elements should be present in a mentoring relationship (Peake & Kelly 2016:19). Although these terms are interchangeably used, they have different meanings as is briefly described below.

*Assessing* is a critical element of mentoring, which involves the assessment of students in clinical practice by specially trained mentors (Douglas et al. 2016:34; RCN 2017). *Supervising* in the form of clinical supervision is a formal process, which involves the guidance and monitoring of trainee practice nurses to promote their independence (Moxham & Gagan 2015:36). Similarly, *preceptoring* is a teaching and learning process that facilitates preceptees or newly qualified registrants to achieve new knowledge, skills and attitudes with support from experienced preceptors in clinical practice (Miller, Vivona & Roth 2016:2015). Finally, *coaching* is a form of learning support that attempts to empower the trainee to adapt to professional changes (Walker-Reed 2016:43).

The effectiveness of mentoring still raises concerns in both developed and developing countries (RCN 2015; Schwerdtle, Morphet & Hall 2017:78). For instance, the Nursing and Midwifery Council (NMC) of the United Kingdom has recently announced significant changes to education standards in replacement of the traditional mentorship model (Duffy & Gillies 2018:18). Clinical mentoring is not a common practice in resource-limited developing countries; if it is practised, it is often informal, infrequent and largely unsupported (Lescano et al. 2019:3). Existing approaches are generally geared towards high-income settings without considering the difference in resources, culture and health structures of resource-limited settings.

### Factors influencing clinical mentoring in nursing

Many studies (Al-Hamdan et al. 2014:248–256; Anarado, Agwu & Nwuno 2016:1–20; De Abreu and Interpeler 2015:42–46) have focussed on the factors influencing mentoring in nursing. Al-Hamdam et al. (2014:249) reported that students identified the relevant knowledge and skills of mentors as being the most important qualities. The authors also emphasised that mentors’ clinical teaching expertise is vital for quality mentoring of students during their practice placement. De Abreu and Interpeller (2015:45) also found that students’ concerns are related to three areas of mentoring, namely the mentor’s qualities and ability to facilitate learning, the mentoring relationship and decision-making in complex situations, as well as support and positive feedback. Likewise, Rylance et al. (2017:407) noted that assessing and supporting students in achieving their goals and developing competencies are key factors that need to be considered during the mentorship.

The mentor’s engagement in the mentoring process is critical. In a systematic review of qualitative studies, Peake and Kelly (2016:18) reported that mentors should facilitate the professional integration of students within their clinical placements, provide the right experience and instil professional attitudes. Setati and Nkosi (2017:136) found that responsive feedback from trained mentors, adequate resources and mutual responsibility contribute to promoting effective mentoring and reducing the theory–practice gap. Clinical settings should meet the requirements for the practice placement of students and mentors should perform according to professional standards, and share their knowledge and experiences to facilitate the integration of theory into practice. Mentors’ roles appear multifaceted, and their personal qualities and characters are the hallmark of mentoring (Sabog, Caranto & David 2015:5).

### Nursing training in Mauritius

Mauritius was a British colony until 1968 and is now an autonomous republic. The training of nurses in the local context has thus been based on the British apprenticeship model. In 2000, the UK phased out the apprenticeship model of training for nurses (NMC 2008), yet student nurses currently still receive ‘on the job’ practical training although they form part of the remunerated workforce. Until 2013, nurses were trained and awarded a Certificate in General Nursing, but thereafter the training moved to a Diploma in General Nursing (DGN).

Training is facilitated by the Central School of Nursing (CSN). The institution, dating to 1958, is under the control of the Ministry of Health and Quality of Life (MOH & QOL). It does not have the status of a college and is affiliated neither to a higher education institution nor to a university. Students undergo practical training in regional hospitals as planned and coordinated by the CSN. The shift from the certificate to DGN was inevitable as it was an old curriculum that has not been reviewed during the past three decades. Furthermore, the old curriculum was no longer responding to the emerging needs of the profession regarding training and education. Consequently, the content was updated and revamped to keep it current with global ongoing changes affecting the nursing profession. During the 3-year DGN training period, students spend 50% of their time on the theoretical and the other 50% on the practical component in clinical settings. The Nursing Council of Mauritius requires that all teaching hospitals be equipped to meet the needs for clinical training with specific reference to ‘mentors with specialised qualifications’ (Nursing Council of Mauritius 2010:6).

Despite having moved to DGN, the approach of supporting students through mentoring in the clinical setting has remained the same. Qualified and experienced nurses carry out the clinical mentoring of students during their practice placement as part of their professional duties. This involves guiding and supervising students whilst they are performing nursing procedures, but without much emphasis on teaching and assessing of theoretical knowledge. The clinical mentoring of students is, therefore, service-led rather than educationally driven. Although the nurses in training hospitals are qualified (as nurses), they are not trained as educators or mentors. Given the above, it is unclear how these qualified nurses experience the clinical mentoring of student nurses in a resource-limited setting.

## Research methods and design

### Design

A qualitative, exploratory, descriptive research approach was used in this study, as it attempted to explore and describe the ideas, thoughts and experiences of qualified nurses whilst mentoring student nurses in the clinical environment with constrained resources (Grove, Gray & Burns 2015:165). This is also referred to as being interpretive because an attempt is made to give meaning to concepts expressed by the qualified nurses (Durand & Tracey 2014:44).

### Research setting

The study took place in Mauritius, an island situated in the south-west of Africa, East of Madagascar in the Indian Ocean. There are five public regional hospitals across the island and other speciality hospitals through which free health service is provided to the public. Some medi-clinics and community health centres also form part of the primary healthcare system. There are two private hospitals and five private clinics. The training of nurses is undertaken by the CSN, which is under the aegis of the MOH and QOL. There is also a private institution that provides training for nurses.

### Population and sample

The research population included qualified nurses working within the five public regional hospitals across the island. According to the register of the CSN, the population for all qualified nurses was *N* = 996. A purposive sample (Gentles et al. 2015:1778) was preferred. The participants were handpicked by the researcher because of their knowledge and experience of clinical mentoring nursing students (Denscombe 2014:50). To be included in the study, qualified nurses needed to have completed their top-up diploma programme meaning they have improved their qualifications from a certificate in nursing to a diploma in nursing studies.

The final sample consisted of eight nurses of which five were male and three, female. A greater number of wards and other units in the regional hospitals are male wards. However, the staffing ratio regarding male nurses to female nurses is almost 1:1 (MOH & QL 2016). Of the eight participants, all were graduates, with five of them holding the top-up DGN, and three were degree holders in nursing. Most of the participants had been in the service for more than 25 years and were posted in general wards such as medical, surgical, gynaecology and orthopaedic. Three were working in speciality units such as intensive care units (ICUs) and operating theatres (Table 1).

**TABLE 1:** Participant profile and codes.

Participant code	Gender	Posting	Qualification	Experience
PA-001	Male	ICU	Top-up Diploma	33 years
PB-002	Male	Theatre	Top-up Diploma	26 years
PC-003	Female	Orthopaedic	Top-up Diploma	20 years
PD-004	Female	ICU	Top-up Diploma	22 years
PE-005	Female	Gynae	Top-up Diploma	28 years
PF-006	Male	Medical	BSc (Hons) Nursing	30 years
PG-007	Male	Surgical	BSc (Hons) Nursing	32 years
PH-008	Male	Orthopaedic	BSc (Hons) Nursing	33 years

ICU, intensive care unit.

### Ethical considerations

Ethical clearance for this study was obtained from the Research Ethics Committee, Department of Health Studies of the University South Africa (HSHDC/539/2016) and the National Research and Ethics Committee of the MOH and QOL (dated 27/03/2017). Participants were provided with a consent or information sheet that explained the full nature of the study. They were also informed that they had the right to withdraw from the study at any point without penalty and that confidentiality and anonymity will be maintained. Those who accepted the terms were then requested to sign the consent or information form.

### Data collection

Data were collected by the first author (DF) by using face-to-face interviews (Grove et al. 2015:83), which were conducted between April and August 2017 in the ward manager’s office. The interviews consisted of one grand tour question namely ‘tell me about your experience of mentoring nursing students in the clinical settings’. Every question was followed up with probes, summaries, clarifications and reflection on the content. With permission from participants, the interviews were audio-recorded and lasted for 25–30 min. A pilot interview was conducted with two participants. The question was found to be clear and understandable. The interviews were added to the final analysis. A total of eight interviews were completed with data saturation perceivably occurring after interview six. Two additional interviews were conducted to confirm the themes and depth of information. Each participant received a code (e.g. PA-001) to preserve their anonymity.

### Data analysis

Data were analysed by using a thematic analysis approach. Interviews were transcribed verbatim by DF. The study involved the identification, reporting and analysis of patterns or themes within the data according to the six steps of *familiarising with data (1), generating initials codes (2), searching for themes (3), refinement of themes (4), defining and naming of themes (5) and producing the report (6)*, as advocated by Braun and Clarke (2006:83). The second author (JM) received a clean set of transcribed data and independently coded the above. The two authors held a consensus discussion and they agreed on the final themes.

### Measures of trustworthiness

The trustworthiness of this study was based on the four key criteria, namely credibility, dependability, confirmability and transferability (Creswell & Creswell 2018:266). Credibility was maintained through prolonged engagement; the researcher spent 6 months in the field. Data were analysed independently by both authors and a peer review was conducted by presenting the findings to three senior members of staff who were not interviewed. Dependability was ensured through the detailed description of the methods. Confirmability was established during ongoing analysis through member checking after confirming with participants the correctness of their responses. Transferability was facilitated by providing in-depth details of all methodological processes.

## Findings and discussions

Table 1 shows an overview of the participant codes, gender, posting, qualification and years of experience.

The findings showed that mentoring *per se* was not practised in this setting. This resulted in the practice being less effective for its original purpose. Possible explanations included a lack of policy directives. Additionally, the mentoring practice was informal with unclear role expectations. Poor material and personal resources further compounded the challenges. An evident absence of buy-in and involvement of management along with a lack of monitoring clinical mentoring by the CSN concludes the picture.

Participants provided several prerequisites needed for clinical mentoring to be successful. As shown in Table 2, this included the need for policy directives for all stakeholders, collaboration between stakeholders, training of mentors, clinical setting requirements and role clarifications.

**TABLE 2:** Summary of main themes.

Themes	Key sub-themes
Mentoring *per se* was not practised, resulting in the current practice being less effective for (its) purpose	Current activities are informal
	Lack of policy directives and standards
	Unclear role expectations
	Poor resources, human and other
	Absence of buy-in from management
	Monitoring students by the CSN
Recommendations for clinical mentoring	The need for policy and directives for all stakeholders
	Collaborations between stakeholders
	Training of mentors
	Clinical setting requirements

CSN, Central School of Nursing.

### Mentoring *per se* was not practised, resulting in the current practice being less effective for (its) purpose

Participants expressed that the current mentoring system did not reflect formal mentoring according to a set definition, but could rather be defined as general support, supervision or coaching. The support seemed to have remained static despite the changes implemented in nursing training since 2013:

‘I do not think mentoring of students is done in this way if someone looks at the definition of a mentor, according to me qualified nurses in the local context cannot be considered as mentors’. (PD-004)

‘[*B*]ut as a qualified nurse, I have to support students when they are posted in the wards.… The support which I give to students is something that has been routinely practiced by other nurses … nothing has changed’. (PF 006)

‘[*A*]nd all through their placement they are guided, supervised and coached by experienced nurses’. (PH-008)

Among the key factors that contributed to the current form of support are those related to the lack of specific policy directives in the wards:

‘But I must say there has never been a clear policy in the wards how to support students who are on placements’. (PD-004)

The approach to support is also informal and there seems to be a disjuncture with what is taught in the nursing school. This is exasperated by the lack of formal training of the nurses in (nursing) education and unclear role expectations. Some nurses felt that their role as mentors were un- or under-recognised:

‘I think this cannot be compared to what is taught at school … as it is very informal. I must also say that as a qualified nurse I am not trained in teaching’. (PH-008)

‘Once again the role of the mentor in the local context is not clear as it is not a post in the local context’. (PC-003)

‘[*A*]s a nurse, I wish I could bring my contribution about what a mentor does, but unfortunately, I will never be recognised as a mentor’. (PA-001)

There are typical constraints as in a resource-poor setting, such as staff shortages, a lack of equipment, overcrowded wards and poor collaboration amongst staff:

‘… many constraints like frequent staff shortage, lack of equipment … overcrowded wards and poor collaboration among staff’. (PB-002)

Not only was there a lack of physical resources, but the nurses seemed to be depleted in terms of their personal resources to support students because of the primary focus being on patient care:

‘I must say that it is difficult for me to give the maximum of myself to support student learning … firstly to provide care as a nurse to patients and secondly to guide and coach students’. (PD-004)

The situation was further exasperated by a lack of buy-in or attention from management:

‘I have noted that mentoring is given less importance because it appears that management does not care about how students’ placement is going’. (PE-005)

‘…I think the management also does not give due attention to mentoring in the ward’. (PG-007)

The lack of monitoring students by the CSN was also viewed as a major shortcoming:

‘[*W*]hat the students do during their placement is not monitored by the school of nursing’. (PB-002)

‘[*A*]nd neither there is monitoring from school regarding the theory–practice gap’. (PH-008)

### Recommendations for clinical mentoring

Participants provided some suggestions on how the current clinical mentoring practice may be improved. They were resolute that clear protocols needed to be in place between the relevant stakeholders, namely the CSN, management and the regulatory body. This included the fulfilment of needs conducive to a learning environment. In addition to the protocols, a need for monitoring was suggested:

‘[*T*]here must be an agreed protocol between the school, the management and the regulatory body’. (PD-004)

‘To me there must be a clear protocol what a learning environment needs, and this must be monitored to keep its standards’. (PD-005)

Participants felt that collaboration among key stakeholders was important to sustain an effective clinical mentoring system:

‘I believe it will be important to have the collaboration of the training institution, the nursing trade unions and the regulatory body to decide on a mentoring system which is adaptable to our context’. (P-007)

The need to train mentors was raised by nearly all participants. They also mentioned some fundamental qualities such as communication skills, teaching and assessment and management or leadership skills:

‘In fact, those who are involved in mentoring should have special training in mentoring and hold a relevant qualification.… One of the fundamental qualities of the mentor is teaching with good communication skills’. (PA-001)

‘I think the central focus must be on teaching, assessing and understanding the content of curriculum … be clinically skilful … teaching skills will matter a lot’. (PD-004)

‘[*T*]hey should have leadership and managerial skills to solve students’ problems’. (PB-002)

The clinical setting was recognised as an essential element that shapes the professional growth of both students and staff:

‘It is the clinical setting which provides opportunities for both students and staff to learn and grow professionally’. (PD-004)

Clinical settings that are poorly equipped are likely to impact on both patients’ care and students’ learning. One participant notably concluded that:

‘[*M*]entors should be allocated enough time to support students … I also think that there must be sufficient equipment, adequate staffing and other learning resources’. (PF-006)

Concerns were raised by participants regarding a pertinent point common to both students and mentors, namely the mentor–student ratio:

‘[*I*]t is also important to decide on the number of students a mentor can take under his responsibility … that is the mentor–student ratio’. (PG-007)

‘[*I*]t must be clear how many students a ward can accommodate, and the student–mentor ratio must be decided so that proper attention can be given to all the students’. (PD-004)

## Discussion

The findings confirm that the lack of a universal description of what clinical mentoring is (Matin 2017:1) results in clinical mentoring being perceived and practised differently across settings (Brand 2016:2). Unclear expectations are exasperated by the lack of policy or protocol directives regarding mentoring in the local context, leading to uncoordinated efforts and support from stakeholders. Standards regarding the practice of clinical mentoring can only be maintained if it is regulated (Rajeswaran 2016:1).

The absence of a clear protocol regarding the ratio of mentors to students indicates that the mentor must support students in the ward irrespective of the numbers. This poses a problem, particularly when one mentor must manage and give individual attention to the students. Although the NMC (2008) advocates a ratio of 1:1, it is now considering reviewing this model of mentoring with less focus on the ratio 1:1 (RCN 2015). The ratio is likely to be influenced by the ward capacity as well as the number of mentors and students posted in the clinical learning environment. In other words, it will be contextually dependant on available resources. In other European countries (such as Belgium, Cyprus, Finland, Ireland, Italy, the Netherlands, Spain, Sweden and the United Kingdom), mentors are exclusively posted in wards to support students, and the ratio is usually 1:5 (Papastavrou et al. 2016:45).

As the mentoring activities were performed informally, it could be perceived as being unofficial. Oluchina and Amayi (2016:179) found that most mentees preferred formal mentoring to informal mentoring, as formal mentoring motivated students to learn and promoted confidence, contrary to informal mentoring. However, the authors also pointed out that informal mentoring has its merits when the students show eagerness to learn and progress.

The training of students is not solely dependent on the mentors in clinical settings, but more importantly, supervision and coordination from the training institution are important for an effective training system. Suitable coordination between training institutions and practice settings is likely a key measure that gives direction to mentoring (RCN 2015). Mwale and Kalawa (2016:1) suggest that the bridging of the theory–practice gap in clinical placements could equally be best addressed through collaboration between the mentor and the educators. Muthati, Thurling and Armstrong (2017:1), for example, recommend that there should always be resource persons from the training institution to monitor and supervise the quality of the students’ clinical placement.

It is important to consider those factors that lead to frustration among mentors, which can negatively impact the quality of mentoring (Rylance et al. 2017:408). This commonly includes a lack of resources, work pressure and poor collaboration. Moreover, career progression, structural changes of workload and the training needs of mentors are just as important in sustaining a successful mentoring system (Peiser et al. 2018:2).

Trained mentors are pivotal to any mentoring system, as Douglas et al. (2016:37) put it. The authors reported that mentors require support in terms of training and partnership with the training institution to better meet the needs of the students. The training of mentors has also been found to have a positive impact on students, nurses and the organisation if there is a rigorous selection among nurses and adequate training (Zhang et al. 2014:136). Teaching and assessing in CLE have been identified as core skills among mentors (De Abreu & Interpeller 2015:45), whereas mentorship programmes require a multitude of teaching strategies, frequent updates and long-term development (Chen & Lou 2014:442).

Although the literature on mentoring does not mention leadership as a quality, it would seem instrumental for the profile of the mentor, owing to their broad responsibilities. The statements on leadership correspond with the findings of Papastavrou et al. (2016:45), which also reported that the leadership style of key stakeholders is an influential factor in mentoring. This view is also upheld by the RCN (2015) in its report, which highlighted that strong leadership is required for mentorship to face current challenges at all levels. The implementation of a training programme for mentorship can however be challenging owing to certain factors, such as lack of organisational support, the absence of a learning culture and unavailability of trainers, as reported by Nowell et al. (2017:1).

### Strengths and limitations

The mentoring system in the local context of Mauritius has remained static for decades. This study provided an updated review of the phenomenon of clinical mentoring in a resource-limited setting. Although the study was conducted in five regional hospitals in Mauritius where the participants were providing clinical support to students, its findings cannot be generalised to other clinical settings. Data were not collected from nurses working in private and specialised hospitals. Despite these limitations, the findings of this study reflect, to a great extent, what other international studies have uncovered on mentoring.

### Implications and recommendations

Given that 50% of the nursing curriculum is dedicated to practice training, there must be adequate coordination from all stakeholders for positive clinical mentoring outcomes. To give clear direction to clinical mentoring, there should be an agreement amongst concerned stakeholders in the form of a policy regarding the learning support of students. Such a policy should spell out the role, responsibilities and firm engagement for stakeholders’ full commitment to sustaining quality mentoring in clinical settings.

Mentoring will continue to be a major challenge in the local context with the advent of the new DGN programme, future reform in training and nursing education and challenges regarding limited resources. From a methodological perspective, it would be interesting to explore the perception of nurse educators on clinical mentoring in the local context using an appreciative inquiry approach. This approach could illuminate the positive aspects and core of ‘what works in organisations and people’ (Hung 2017:1).

## Conclusion

Clinical mentoring as a phenomenon was studied in five regional hospitals in Mauritius. Although mentoring is differently exercised across clinical settings, the basic principles should at least be observed despite there being no universal standards for clinical mentoring. What constitutes standards of practice for clinical mentoring needs to be debated and agreed by academics, regulatory bodies, practitioners and professional nurses’ associations. The ever-changing healthcare environment directly affects the suitability and adaptability of clinical placements in relation to clinical mentoring, and the CLE remains at the epicentre of clinical mentoring. It is therefore fundamental for all stakeholders with an interest in nursing to collaboratively strive to have a better understanding of and recognise that clinical mentoring is an important tool that will help in providing the next generation of competent nurses who are fit for practice in complex healthcare environments.
